# Evaluating the One-Year Impact of School e-Cigarette Use Interventions among Current Youth e-Cigarette Users in the COMPASS Study, 2017/18–2018/19

**DOI:** 10.3390/ijerph20146353

**Published:** 2023-07-13

**Authors:** Adam G. Cole, Mahmood R. Gohari, Scott T. Leatherdale

**Affiliations:** 1 Faculty of Health Sciences, Ontario Tech University, Oshawa, ON L1G 0C5, Canada; 2 School of Public Health Sciences, University of Waterloo, Waterloo, ON N2L 3G1, Canada; mgohari@uwaterloo.ca (M.R.G.);

**Keywords:** adolescent, e-cigarette use, prevention, cessation, evaluation, natural experiment

## Abstract

There is a lack of evidence for the impact of school-based e-cigarette interventions among current e-cigarette users. This natural experimental evaluation study evaluated the one-year impact of various school-based e-cigarette prevention/cessation programs among a sample of current youth e-cigarette users. The COMPASS study sample included *n* = 3586 current e-cigarette users from *n* = 90 schools with data collected between 2017 and 2019. Student e-cigarette use patterns were categorized as “escalated”, “maintained”, and “reduced” based on the change in past 30-day e-cigarette use between baseline and follow-up. Intervention schools added e-cigarette use “prevention”, “cessation”, or “protection” programs, while control schools did not make any changes. Logistic regression models identified how each category of added programs was associated with e-cigarette use patterns. About one quarter of schools added an e-cigarette use prevention/cessation program over one year. Student e-cigarette use patterns between control and intervention groups differed in proportion ranging from a decrease of 3.35% to an increase of 5.80%. Regression models did not identify any significant differences in the odds of escalating or reducing e-cigarette use in intervention relative to control schools. While many schools implemented new e-cigarette programs over one year, none of the interventions led to significant changes in e-cigarette escalation or reduction among current youth e-cigarette users. Additional studies are needed to evaluate the impact of e-cigarette interventions among current e-cigarette users.

## 1. Introduction

It is well-known that e-cigarette use (or vaping) is a prevalent concern among youth in Canada and the United States [1,2]. The school environment is an important setting where youth from a variety of backgrounds spend a significant amount of time and where they can be influenced by programs, policies, and peers [3,4,5,6]. While there is evidence that schools are implementing prevention and cessation intervention programs to reduce e-cigarette use [7,8], due to the novelty of these products, there are few evaluations of school-based e-cigarette interventions [9,10,11,12,13]. While some studies have focused on evaluating whether these interventions prevent e-cigarette use initiation [11,13], to our knowledge, there are no evaluations of school-based e-cigarette use interventions among current e-cigarette users, including whether cessation interventions encourage students to quit or reduce their e-cigarette use frequency. School-based e-cigarette interventions may result in students maintaining, escalating, or reducing their frequency of e-cigarette use. In addition, school-based prevention programs are typically delivered to all students in a school or classroom, regardless of their e-cigarette use status. Therefore, students who use e-cigarettes are also exposed to the information presented in prevention programs, which may influence their decision to change their e-cigarette use pattern. Evaluations of prevention programs usually only examine whether these programs delay or prevent the initiation of substance use. We believe that examining the impacts of prevention programs among current users provides important information about potential unintended impacts (either positive or negative) of this programming. Most other studies also evaluate researcher-initiated interventions that are highly controlled; schools may choose to implement e-cigarette interventions on their own (a type of natural experiment), and these types of interventions do not typically get evaluated. Therefore, the objective of this quasi-experimental study was to evaluate the one-year impact of a variety of school-based e-cigarette interventions among a sample of current youth e-cigarette users.

## 2. Materials and Methods

This natural experimental evaluation study used two years of linked longitudinal data from a convenience sample of secondary schools and students participating in the CIHR-funded COMPASS study [14]. The COMPASS study is a prospective cohort study that collects data annually from a convenience sample of students and the schools they attend in four Canadian provinces [14]. It is designed to evaluate how changes in school policies, programs, and the built environment affect a variety of youth health behaviors [14]. The COMPASS study received ethics approval from the University of Waterloo Research Ethics Board (#30118) and appropriate school board review committees, and this secondary data analysis received ethics approval from the Ontario Tech University Research Ethics Board (#15884).

### 2.1. Participants

This study used data from a sample of past 30-day (current) youth e-cigarette users in grades 9 to 11 (ages 13–17) from four Canadian provinces in Year 6 (2017/18, baseline) and Year 7 (2018/19, follow-up) of the COMPASS study. Consistent with a previous study [13], we excluded schools (*n* = 20) that removed an e-cigarette use prevention/cessation program between baseline and follow-up and schools (*n* = 7) that had student-level data but no school-level data. At baseline, *n* = 7712 students in grades 9–11 (secondary III–IV in Quebec) attending *n* = 90 secondary schools across Ontario (*n* = 46), Quebec (*n* = 26), British Columbia (*n* = 11), and Alberta (*n* = 7) reported using e-cigarettes in the last 30 days. Students who could not be linked at follow-up (*n* = 4126) were removed from the sample, leaving a final linked sample of *n* = 3586 current e-cigarette users (3586/7712 = 46.5% of baseline current e-cigarette users). The sample of current e-cigarette users was approximately half male (54.3%), and the majority were white (76.8%).

### 2.2. Measures

Student-level data were collected during class time using the COMPASS questionnaire [15]. At the time of the survey, the questionnaire referred to vaping devices as “e-cigarettes” and did not include a definition or examples of devices. Students reported the number of days in the last 30 days that they used e-cigarettes [response options: none, 1 day, 2 to 3 days, 4 to 5 days, 6 to 10 days, 11 to 20 days, 21 to 29 days, and 30 days (every day)]. Based on responses to this question at baseline and follow-up, current e-cigarette users who reported “escalated” use increased the number of days they used e-cigarettes between baseline and follow-up, those who reported “reduced” use decreased the number of days they used e-cigarettes between baseline and follow-up (including not using e-cigarettes at all at follow-up), and those who reported “maintained” use used e-cigarettes the same number of days at baseline and follow-up. Students also reported their baseline grade (9, 10, 11), gender (female, male), self-reported ethnicity (white, non-white), amount of weekly spending money, cigarette smoking status, and cannabis use status.

Classification of schools into control and intervention groups was accomplished using data from the School Policies and Programs questionnaire (SPP). The SPP is completed annually by a school contact knowledgeable about the school’s health-related programs and policies [14]. As described elsewhere [13], school contacts were asked whether the school offered any programs (other than classes/curriculum) that addressed e-cigarette use prevention, tobacco use prevention, or tobacco use cessation and whether these programs were new or continuing from previous years. Tobacco use prevention and cessation programs were included in this study to be as comprehensive as possible given that some respondents may include e-cigarette prevention or cessation programs as part of tobacco programming. Data on school neighborhood median income were collected using data from the 2016 census. Consistent with previous studies [4], we also calculated the baseline senior student (grade 11 in Quebec, grade 12 in all other provinces) e-cigarette use rate for each school as a proxy for the social environment for e-cigarette use within a school.

### 2.3. Analysis

As described elsewhere [13], we identified schools that added new e-cigarette/tobacco use prevention/cessation programs between baseline and follow-up and categorized the programs based on the Smoke-Free Ontario (SFO) four pillars of tobacco control: “industry” (e.g., interventions to counter the tobacco industry’s efforts to sell products), “prevention” (e.g., interventions to prevent e-cigarette initiation), “protection” (e.g., interventions to protect against exposure to second-hand vapor), and “cessation” (e.g., interventions that encourage quit attempts) [16]. School interventions were grouped in this way to increase the sample size for analysis. Consistent with recommendations for evaluating natural experiments [17], schools that did not add a new program in 2018/19 were categorized as control schools. We identified the percentage of current e-cigarette users who escalated, reduced, and maintained e-cigarette use in intervention schools relative to control schools. Separate generalized estimating equation (GEE) logistic regression models identified how each category of added programs was associated with the pattern of escalating or reducing e-cigarette use (relative to maintaining e-cigarette use) while accounting for the nesting of students within schools and controlling for baseline student gender, grade, ethnicity, amount of spending money, cigarette smoking, and cannabis use, and school baseline neighborhood median income, school-level senior e-cigarette use rate, and province. As a sensitivity analysis, we examined whether the results changed when we excluded schools from the control group that reported e-cigarette/tobacco use prevention/cessation programming at baseline (*n* = 23) and when we excluded schools in the intervention group that reported e-cigarette/tobacco use prevention/cessation programming at baseline (*n* = 13). Statistical analyses were conducted using SAS software, Version 9.4 [18].

## 3. Results

As previously reported [13], at one-year follow-up, *n* = 24 schools added an e-cigarette/tobacco use prevention/cessation program (*n* = 7 in Quebec, *n* = 14 in Ontario, *n* = 3 in British Columbia), and four of these schools reported adding more than one type of program (e.g., both prevention and cessation program). Based on the descriptions of interventions provided and SFO classifications, *n* = 19 schools added prevention programs, *n* = 3 added protection programs, and *n* = 6 added cessation programs. Eight schools indicated adding prevention and/or cessation programs but did not provide any details about what this programming included. Prevention programs included tobacco-free or addiction prevention theme weeks (*n* = 2), an externally developed, interactive presentation about the harms of vaping (*n* = 4), teacher-developed lessons about the harmful effects of vaping (*n* = 1), guest presentations about substance abuse (*n* = 2), parent information nights and discussions about tobacco use (*n* = 1), and tobacco use workshops hosted by a special education technician (*n* = 1). Protection programs included presentations to students about the laws around vaping (*n* = 1), a suspension re-entry program for vaping (*n* = 1), and presentations to students about fines for vaping and smoking on school property (*n* = 1). Cessation programs included a school-developed cessation program (*n* = 1), cessation information provided by a school nurse or Tobacco Enforcement Officer (*n* = 2), and a quit contest (*n* = 1). There were some small differences in the demographic characteristics of students in the control and intervention groups (Table 1); therefore, these characteristics were included in subsequent regression analyses.

Table 2 presents the percentage of current youth e-cigarette users reporting each e-cigarette use pattern in control and intervention schools. The difference in the percentage of e-cigarette use patterns between the control and intervention schools was relatively small and ranged from a decrease of 3.35% to an increase of 5.80%. The largest difference was evident for schools that added a protection program, and the results were in a positive direction (i.e., fewer students who escalated or maintained and more students who reduced e-cigarette use frequency).

After controlling for covariates, GEE logistic regression models did not identify any significant differences in the odds of escalating or reducing e-cigarette use compared with maintaining e-cigarette use among current youth e-cigarette users in intervention schools relative to control schools (Table 3). The results did not change when we excluded schools from the control group that reported e-cigarette/tobacco use prevention/cessation programming at baseline (Table S1) and when we excluded schools in the intervention group that reported e-cigarette/tobacco use prevention/cessation programming at baseline (Table S2).

## 4. Discussion

This evaluation of multiple school-based natural experiments identified that many schools are implementing interventions targeting youth e-cigarette use. However, these interventions only resulted in small changes in patterns of escalating and reducing e-cigarette use among current e-cigarette users. While 36 schools reported already having some kind of e-cigarette/tobacco use intervention in place at baseline, only about a quarter of schools (24/90) implemented a new e-cigarette/tobacco use intervention during a period of time when the e-cigarette use prevalence rapidly increased among youth in Canada [1,2]. This means that about half of schools (90 − 47 = 43) had no e-cigarette/tobacco use programming in place over the two years of study. While few schools implemented a new e-cigarette use intervention, it may be due to a lack of effective and feasible program options. While there are examples of evidence-based e-cigarette prevention programs [9,10,11], there is scant evidence for e-cigarette cessation programs. Given the popularity of vaping among youth and growing concerns about nicotine addiction, such programs are clearly needed. Other data indicate that few teachers in California reported the use of education, alternative-to-suspension, or cessation programs for students caught vaping in class [19].

Our models did not identify any significant impacts over one-year for these school program changes, despite adjusting for a variety of factors. It is possible that due to the small sample size of current e-cigarette users within each group, there was insufficient power to identify significant changes in e-cigarette use patterns, particularly for cessation programs where only six schools added this type of program. In addition, longer follow-up periods may be needed for program impacts to be fully realized, particularly for e-cigarette cessation programs where multiple cessation attempts may be necessary for students to be successful. It is also possible that the interventions may have been missing important components that would increase their effectiveness. Due to the retrospective nature of these analyses, we were unable to follow-up with schools to identify the specific components that were included. Given the importance of evaluating the impact of school-based e-cigarette use interventions among current e-cigarette users, future studies should recruit a sufficient number of schools and students, collect data more frequently and over a longer follow-up period, and collect information about intervention components and fidelity.

The descriptive results suggest that protection interventions may hold the most promise in changing e-cigarette use patterns among current youth e-cigarette users in the desired direction (i.e., fewer students escalating/maintaining e-cigarette use and more students reducing/stopping e-cigarette use). The current natural experimental evaluation included many types of interventions within each group, and the heterogeneity in the categories of added programs may have muted intervention impacts. Within this study, interventions categorized as protection programs included discussions about the laws around vaping, implementing a suspension re-entry program for vaping, and speaking about fines for vaping and smoking on school property. A previous study evaluating the impact of banning the use of e-cigarettes at school (a type of e-cigarette protection program) identified that these policies led to lower odds of current e-cigarette use among students in intervention relative to control schools [12], supporting the potential promise of interventions that focus on protecting students from exposure to e-cigarette use at school.

To our knowledge, this study is the first to evaluate the impact of a variety of school-based e-cigarette prevention/cessation programs on e-cigarette use patterns among current youth e-cigarette users. A key strength of this study is the use of a large, school-based longitudinal data set that includes both intervention and control schools to evaluate multiple natural experiments. Limitations include the lack of a definition of e-cigarettes or examples of common brands in the questionnaire and categorical responses for the frequency of e-cigarette use, which may not represent the usual use pattern of students. The response options for the measure of e-cigarette use frequency are defined categories, and changes in e-cigarette use frequency between baseline and follow-up may not always be captured. Given that some students who escalated or reduced the frequency of e-cigarette use may provide the same response category at baseline and follow-up, our results may underestimate the proportion of youth who escalated and reduced e-cigarette use and overestimate the proportion of youth who maintained e-cigarette use. This may bias the results towards null findings. An additional limitation includes the lack of program detail to know how programs were implemented, what their components were, and to what extent they were evidence informed. Many interventions were categorized as prevention programs, which may have limited impact on current e-cigarette users since the target of these types of programs tends to be students who do not use e-cigarettes; however, school-based prevention programs are usually delivered to all students within a school or classroom, and students who use e-cigarettes may change their e-cigarette use pattern after exposure to these programs. While some schools may have been incorrectly categorized as control schools if they forgot to report a change in the SPP, we expect this would have a minimal effect given the number of control schools included in the study. These analyses only examined a one-year change in e-cigarette use patterns; given that the COMPASS study is ongoing, future studies could evaluate longer-term patterns of e-cigarette use following changes in school programming. Finally, we may not have had sufficient power to identify significant changes in e-cigarette use patterns between intervention and control groups given the smaller sample of current youth e-cigarette users.

## 5. Conclusions

While a few schools implemented new e-cigarette use prevention/cessation programs over one-year, none of the interventions in this natural experimental evaluation led to significant changes in e-cigarette use patterns among current youth e-cigarette users. While interventions that discourage youth from using e-cigarettes at school may hold promise, additional studies are needed to evaluate the impact of such programs among current youth e-cigarette users.

## Figures and Tables

**Table 1:** Baseline demographic characteristics of students and schools in the control and interventions groups, 2017/18–2018/19 COMPASS study.

Baseline Characteristic	Control Group (*n* = 3087) *n* (%)	School Added Prevention Program (*n* = 728) *n* (%)	School Added Cessation Program (*n* = 249) *n* (%)	School Added Protection Program (*n* = 145) *n* (%)
**Student-level Characteristics**				
Grade				
9	809 (31.09)	277 (38.05)	98 (39.36)	52 (35.86)
10	1130 (43.43)	271 (37.23)	101 (40.56)	57 (39.31)
11	663 (25.48)	180 (24.73)	50 (20.08)	36 (24.83)
Gender				
Female	1168 (44.75)	344 (47.25)	126 (50.60)	73 (50.34)
Male	1442 (55.25)	384 (52.75)	123 (49.40)	72 (49.66)
Ethnicity				
White	2000 (76.60)	580 (79.67)	183 (73.49)	106 (73.10)
Non-white	611 (23.40)	148 (20.33)	66 (26.51)	39 (26.90)
Amount of weekly spending money				
≤$20	862 (33.14)	241 (33.29)	104 (42.11)	47 (32.64)
$21–$100	749 (28.80)	202 (27.90)	62 (25.10)	49 (34.03)
>$100	673 (25.87)	194 (26.80)	54 (21.86)	34 (23.61)
I don’t know	317 (12.19)	87 (12.02)	27 (10.93)	14 (9.72)
Cigarette smoking status				
Never smoker	1356 (52.01)	408 (56.12)	153 (61.69)	81 (55.86)
Ever smoker	685 (26.28)	190 (26.13)	57 (22.98)	35 (24.14)
Current smoker	566 (21.71)	129 (17.74)	38 (15.32)	29 (20.00)
Cannabis use status				
Never user	1065 (41.07)	320 (44.26)	115 (46.75)	56 (38.89)
Ever user	751 (28.96)	202 (27.94)	62 (25.20)	54 (37.50)
Current user	777 (29.97)	201 (27.80)	69 (28.05)	34 (23.61)
**School-level Characteristics**				
School neighborhood median income				
$25,001–$50,000	170 (6.51)	78 (10.71)	105 (42.17)	51 (35.17)
$50,001–$75,000	1552 (58.29)	460 (63.19)	144 (57.83)	94 (64.83)
$75,001–$100,000	767 (29.38)	190 (26.10)	0.0 (0)	0.0 (0)
>$100,000	152 (5.82)	0.0 (0)	0.0 (0)	0.0(0)
Mean school-level e-cigarette use at baseline (stdev)	26% (7%)	27% (7%)	25% (9%)	29% (7%)
Mean school-level senior student e-cigarette use rate (stdev)	30% (8%)	33% (9%)	32% (10%)	37% (10%)

**Table 2:** Percentage of current youth e-cigarette users reporting each e-cigarette use pattern in the group of control and intervention schools, 2017/18–2018/19 COMPASS study.

School Intervention Classification	Maintained e-Cigarette Use		Escalated e-Cigarette Use		Reduced e-Cigarette Use	
	*n* (%)	Diff ^1^	*n* (%)	Diff ^1^	*n* (%)	Diff ^1^
Control group	460 (17.62)	-	1276 (48.87)	-	875 (33.51)	-
School added any program	181 (18.39)	+0.77	473 (48.07)	−0.80	330 (33.54)	+0.03
School added prevention program	139 (19.09)	+1.47	355 (48.76)	−0.11	234 (32.14)	−1.37
School added cessation program	39 (15.66)	−1.96	124 (49.80)	+0.93	86 (34.54)	+1.03
School added protection program	22 (15.17)	−2.45	66 (45.52)	−3.35	57 (39.31)	+5.80

^1^ Diff is the difference in the percentage of students in each e-cigarette use pattern between the group of intervention and control schools.

**Table 3:** Odds of escalating and reducing e-cigarette use among the sample of current youth e-cigarette users in the group of intervention schools compared with control schools, 2017/18–2018/19 COMPASS study.

School Intervention Classification	Odds of Escalating e-Cigarette Use (vs. Maintaining) ^1^ OR (95% CI)	Odds of Reducing e-Cigarette Use (vs. Maintaining) ^1^ OR (95% CI)
Control group (reference)	1.00	1.00
School added any program	0.93 (0.74, 1.16)	0.97 (0.73, 1.30)
School added prevention program	0.89 (0.68, 1.16)	0.90 (0.67, 1.22)
School added cessation program	1.28 (0.83, 1.99)	1.13 (0.69, 1.89)
School added protection program	1.03 (0.59, 1.80)	1.52 (0.76, 3.02)

^1^ Models controlled for baseline student gender, grade, ethnicity, amount of spending money, cigarette smoking, and cannabis use, and school baseline neighborhood median income, school-level senior e-cigarette use rate, province, and student-level clustering within schools.

## Data Availability

COMPASS data is stored at the University of Waterloo on a secure server. The principal investigator of COMPASS, S.T.L., maintains ownership of all COMPASS data and will grant access to COMPASS research collaborators, external research groups, and students. For researchers looking to gain access to COMPASS data, individuals must successfully complete the COMPASS data usage application form that is available online (https://uwaterloo.ca/compass-system/information-researchers, accessed on 12 May 2023), which is then reviewed and approved/declined by STL.

## Supplementary Materials

The following supporting information can be downloaded at: https://www.mdpi.com/article/10.3390/ijerph20146353/s1, Table S1. Sensitivity analysis examining the odds of escalating and reducing e-cigarette use among the sample of current youth e-cigarette users in the group of intervention schools compared with control schools, excluding schools from the control group that reported baseline e-cigarette/tobacco use prevention/cessation programming, 2017/18–2018/19 COMPASS study; Table S2. Sensitivity analysis examining the odds of escalating and reducing e-cigarette use among the sample of current youth e-cigarette users in the group of intervention schools compared with control schools, excluding schools from the intervention group that reported baseline e-cigarette/tobacco use prevention/cessation programming, 2017/18–2018/19 COMPASS study.
