# Supplementary material for: Evaluating the One-Year Impact of School e-Cigarette Use Interventions among Current Youth e-Cigarette Users in the COMPASS Study, 2017/18–2018/19
Source: Int J Environ Res Public Health. 2023 Jul 13;20(14):6353. doi: 10.3390/ijerph20146353 (PMC10379233; doi:10.3390/ijerph20146353)
Supplement: Supplementary file 1 [file ijerph-20-06353-s001.zip › ijerph-2421988-supplementary.pdf]

Table S1. Sensitivity analysis examining the odds of escalating and reducing e-cigarette use among the sample of current youth e-cigarette users in the group of intervention schools compared with control schools, excluding schools from the control group that reported baseline e-cigarette/tobacco use prevention/cessation programming, 2017/18-2018/19 COMPASS study

School intervention classification	Odds of Escalating e-Cigarette Use (vs Maintaining) [1] OR (95% CI)	Odds of Reducing e-Cigarette Use (vs Maintaining) [1] OR (95% CI)
Control group (reference)	1.00	1.00
School added any program	0.98 (0.75, 1.29)	1.01 (0.74, 1.37)

[1] Models controlled for baseline student gender, grade, ethnicity, amount of spending money, cigarette smoking, and cannabis use, and school baseline neighbourhood median income, school-level senior e-cigarette use rate, province, and student-level clustering within schools.

Table S2. Sensitivity analysis examining the odds of escalating and reducing e-cigarette use among the sample of current youth e-cigarette users in the group of intervention schools compared with control schools, excluding schools from the intervention group that reported baseline e-cigarette/tobacco use prevention/cessation programming, 2017/18-2018/19 COMPASS study

School intervention classification	Odds of Escalating e-Cigarette Use (vs Maintaining) [1] OR (95% CI)	Odds of Reducing e-Cigarette Use (vs Maintaining) [1] OR (95% CI)
Control group (reference)	1.00	1.00
School added any program	0.99 (0.71, 1.38)	0.84 (0.55, 1.28)

[1] Models controlled for baseline student gender, grade, ethnicity, amount of spending money, cigarette smoking, and cannabis use, and school baseline neighbourhood median income, school-level senior e-cigarette use rate, province, and student-level clustering within schools.
